# Risk factors associated with prognosis in patients with osteoradionecrosis of the jaws: A single institutional experience over 15 years

**DOI:** 10.4317/medoral.27164

**Published:** 2025-05-27

**Authors:** Chen‑xi Li, Zhong‑cheng Gong, Sakendeke Jumatai, Chang Fang, Parekejiang Pataer, Hua-rong Zhao

**Affiliations:** 1 Assistant professor. Department of Oral and Maxillofacial Oncology and Surgery, School / Hospital of Stomatology, the First Affiliated Hospital of Xinjiang Medical University, Urumqi, China; 2 Laboratory head. Stomatological Research Institute of Xinjiang Uygur Autonomous Region, National Clinical Medical Research Institute, Urumqi, China; 3 Professor, department head. Department of Oral and Maxillofacial Oncology and Surgery, School / Hospital of Stomatology, the First Affiliated Hospital of Xinjiang Medical University, Urumqi, China; 4 Resident. Department of Oral and Maxillofacial Radiology, the First Affiliated Hospital of Xinjiang Medical University, Urumqi, China; 5 Resident. Department of Oral and Maxillofacial Oncology and Surgery, School / Hospital of Stomatology, the First Affiliated Hospital of Xinjiang Medical University, Urumqi, China; 6 Attending doctor, research assistant. Department of Oral and Maxillofacial Oncology and Surgery, School / Hospital of Stomatology, the First Affiliated Hospital of Xinjiang Medical University, Urumqi, China; 7 Professor, department head. Division of Radiation Oncology, Cancer Center, the First Affiliated Hospital of Xinjiang Medical University, Urumqi, China

## Abstract

**Background:**

Osteoradionecrosis of the jaws (ORNJ) is a pernicious complication of radiation therapy that significantly affects the quality of life of patients with head and neck cancer. The present study aimed to investigate the risk factors for the clinical prognosis of ORNJ in the same scenario.

**Material and Methods:**

A cross-sectional study was designed and implemented in a tertiary teaching hospital from January 2005 to December 2020. A total of 106 patients were divided into normal wound healing group (n = 79) and delayed wound healing group (n = 27) according to two different prognosis. The risk factors associated with the prognosis in patients with ORNJ were comparatively analyzed via performing one-way and multifactorial logistic analyses.

**Results:**

The majority of the study cohort (n = 59, 55.7%) was found to be characterized with Glanzmann and Gratz grade 2 and followed up for a median of 38.6 months. Diabetes mellitus (*P* = .045), Charlson comorbidity index (*P* = .042), American Society of Anesthesiologists score (*P* < .001), primary tumour site (*P* = .012), T stage (*P* = .008), ORNJ grade at initial diagnosis (*P* < .001), pan-immune-inflammatory value and systemic immune-inflammatory index at initial radiotherapy (*P* = .01 and *P* < .001 respectively) were detected as risk factors associated with poor prognosis in patients with ORNJ.

**Conclusions:**

We conclude that there are abundant risk factors for poor prognosis in these patients, and it is important to be evaluated before irradiation so that suiTable post-radiated treatments can be given.

** Key words:**Pan-immune-inflammatory value, systemic immune-inflammatory index, jaw, osteoradionecrosis, clinical prognosis.

## Introduction

Head and neck cancer ranked the eighth most common malignant tumour worldwide in 2022 (892,000 new cases and 458,000 deaths), accounting for 7.9% of all malignancies and over 5.1% of all cancer-related mortalities (1). Most head and neck cancers (around 90%) are derived from the mucosal epithelium in the oral cavity, pharynx, larynx, nasal cavity and paranasal sinuses and are known collectively as head and neck squamous cell carcinomas (HNSCCs) (2). Primary or adjuvant radiotherapies, often administered alongside chemotherapy, are essential components of non-surgical treatment protocols (3). Although there have been remarkable advances in radiation therapy (RT) planning and delivery, acute and late toxicities are frequently linked to significant morbidity. The mouth is especially susceptible to adverse complications from irradiation. These include rampant dental caries, oral candidosis, trismus, mucosal irritation or mucositis, dysgeusia or taste changes, xerostomia (salivary gland dysfunction), radiation fibrosis syndrome, and osteoradionecrosis. Such complications significantly detriment oral health-associated quality of life both during and after treatment (3,4).

The majority of head and neck cancer patients are at risk of developing osteoradionecrosis of the jaws (ORNJ). This condition is the most serious complication associated with radiotherapy that often occurs with concomitant tooth mobility and loss, resulting in oromaxillofacial dysfunction and defects, which may greatly reduce the quality of life (5). Previous studies have reported that factors such as dental extraction, radiation field, and tobacco habit history all play an important role in increasing the incidence of ORNJ (5-7). However, to our best knowledge, there are few studies on the outcomes that evaluate the predictive value of various prognostic indexes measured by laboratory test, and these studies generally include small patient populations.

Consequently, this study aimed to investigate the clinicopathological data on patients with ORNJ for use in its prediction and to discuss the epidemiology, etiology, pathophysiological characteristics, and treatment modalities thereof. This may provide a reference point and guide for clinicians, potentially influencing their clinical management planning.

## Material and Methods

- Study design

To achieve the study objectives, the researchers conducted a cross-sectional retrospective study following the Strengthening the Reporting of Observational Studies in Epidemiology (STROBE) guidelines (8). The study population consisted of patients who visited authors’ department for evaluation and management of ORNJ between January 2005 and December 2020. The study protocol was reviewed and approved by the ethics committee of authors’ affiliation (approval no. K20200706-11). Procedures in this research adhered to the standards of the Helsinki Declaration and laboratory regulations in China. All data collected during this study are included in this published article. All participating individuals provided informed consent.

- Patient selection

The diagnosis of ORNJ is mainly based on patient history and clinical findings. The diagnostic key points are listed below: i) ORNJ can manifest itself over a period of 3 months after irradiation of the area and usually starts with a nonhealing ulcer, eventually leading to exposure of necrotic bone. ii) Severe pain and difficulties in chewing or swallowing can result from a secondary infection of the hypoxic tissues. iii) In advanced stages of ORNJ, bone sequestration may occur along with orocutaneous fistula formation, pathological fractures, local or systemic infection, and trismus. iv) The radiographic images reveal that the affected bone exhibits sclerosis, resorption, and periosteal reaction (9).

To be included in this classification, patients had to meet the following criteria: i) Must be in accordance with the definition that radiological evidence indicates bony necrosis within the irradiated region of the jaw (10). ii) Patient characteristics must conform to the aforementioned diagnostic criteria. iii) Patients with primary or recurrent head and neck tumours who received radiotherapy in definite or adjuvant setting with/without chemotherapy. iv) Did not have any antiresorptive drugs before, during or after the treatment procedure.

- Data acquisition

**Figure 1:**
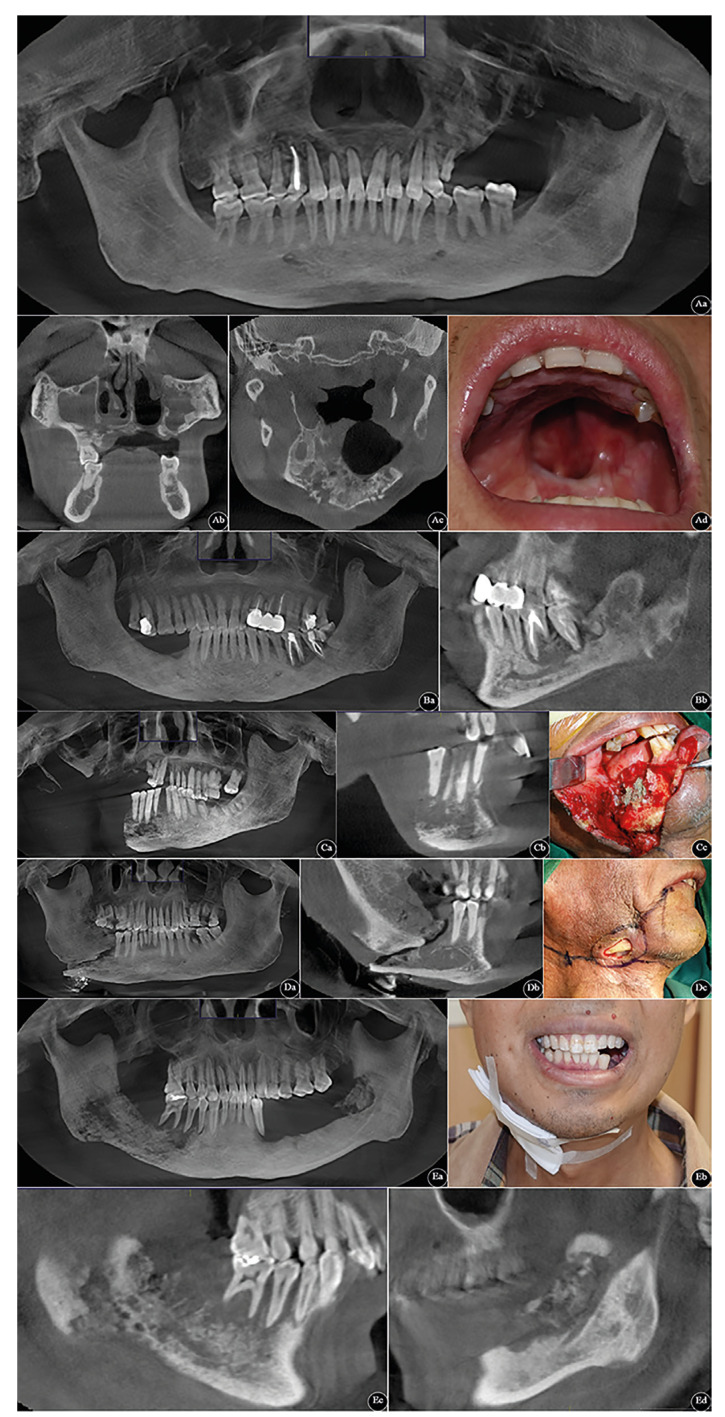
Typical patients’ maxillofacial cone beam computed tomography (CBCT) images are classified according to Glanzmann and Grätz grading system of ORNJ. A-E represents Grade 0 to 4. Grade 0: Aa. panoramic radiograph; Ab. coronal view; Ac. axial view; Ad. intraoral photo. Grade 1: Ba. panoramic radiograph; Bb. sagittal view. Grade 2: Ca. panoramic radiograph; Cb. sagittal view; Cc. intraoperative image. Grade 3: Da. panoramic radiograph; Db. sagittal view; Dc. preoperative image. Grade 4: Ea. panoramic radiograph; Eb. extraoral photograph; Ec. right-side sagittal view; Ed. left-side sagittal view.

The items collected included demographics, habits, general oral health, comorbid factors, extractions, primary tumour site, tumour-node-metastasis (TNM) cancer staging, and seroimmunological indexes [e.g., pan-immune-inflammatory value (PIV), systemic immune-inflammatory index (SII)]. Among them, PIV and SII were calculated using the following formula: PIV = neutrophil counts (×109/L) × platelet counts (×109/L) × monocyte counts (×109/L) ÷ lymphocyte counts (×109/L); SII = neutrophil counts (×109/L) × platelet counts (×109/L) ÷ lymphocyte counts (×109/L). By retrieving for the registered cases in Hospital Information and Management System (HIMS), the total population was categorized according to the adopted modified Glanzmann and Grätz classification system (Fig. 1) (11):

Grade 0 - Radiographic ORNJ with intact mucosa

Grade 1 - Exposed necrotic bone without signs of infection for at least 3 months

Grade 2 - Exposed necrotic bone with signs of infection or sequestrum, but not grade 3 or 4

Grade 3 - ORNJ resulting in pathologic fracture or ORNJ treated with surgical resection, with satisfactory result

Grade 4 - ORNJ refractory to surgical resection

- Statistical analyses

Demographic and clinical data were processed using R packages, version 4.2.1 (R Foundation for Statistical Computing, Vienna, Austria) with odds ratio (OR) being used to compare categorical variables and mean difference for continuous statistics. These data were measured using appropriate effect size and 95% confidence intervals (CIs). Chi-square or Fisher’s exact test were performed for univariate analysis, and a stepwise logistic regression analysis with standardized regression coefficients was employed for multivariate analysis. *P* < .05 (e.g., *P < .05, **P < .01, ***P < .001) was considered statistically significant.

## Results

- General information of study population

A total of 106 patients with ORNJ met the inclusion criteria after carefully screening medical record files in HIMS. These HNSCC cases were treated with radiation therapy in either adjuvant or definitive setting and followed up for a median of 38.6 months. At the time of diagnosis, the majority of the study cohort (n = 59, 55.7%) was found to be characterized with Grade 2 (Glanzmann and Grätz grading) - chronic persistent non‑progressive lesions.

- Demographics and clinicopathological characteristics

Out of 106 ORNJ individuals, 38.7% (n = 41) were female and 61.3% (n = 65) were male. Their ages ranged from 28 to 86 years, with an average age of 66.2 ± 9.6 years. The oral habits in form of drinkless or drunk alcohol before cancer treatment were noted in respectively 67.0% (71/106) and 33.0% (35/106) of the total population, and 37 of 106 (34.9%) patients were former smokers. The general oral health was moderate (mild gingivitis and 2-3 dental caries) in 47.2% (50/106) and poor (multiple carious teeth and periodontal compromised teeth) in 52.8% (56/106) of the study population, respectively. Comorbid factors like diabetes mellitus (n = 12, 11.3%), hypertension (n = 21, 19.8%), chronic obstructive pulmonary disease (n = 6, 5.7%), and thyroid disorders (n = 19, 17.9%) were present (Table 1).

- Tumour-and ORNJ-related information of study population

The primary tumour sites included oral cavity (n = 85, 80.2%), oropharynx (n = 13, 12.3%), nasopharynx (n = 1, 0.9%), and hypopharynx (n = 7, 6.6%). Data concerning on M stage were not shown because no patients had distant metastases at the time of clinical/radiographic examination. The most common disease site in the study population was mandible (n = 97, 91.5%), followed by maxilla (n = 9, 8.5%). Most of the patients presented with Grade 2 ORNJ (55.7%, 59/106) using Glanzmann and Grätz classification system (Table 1). A summary of ORNJ features is presented in Table 1.

- Univariate analysis of risk factors impacting the prognosis in patients with ORNJ

In conformity to patients’ clinical prognosis, the included cases were divided into a normal wound healing (NH) group (within 7 to 10 days postoperatively) and a delayed wound healing (DH) group (over 15 days postoperatively) (12). Univariate analysis revealed that comorbidities such as diabetes, American Society of Anesthesiologists (ASA) score, Charlson comorbidity index (CCI), primary tumour site, T stage, site of ORNJ and its grade at initial diagnosis, and seroimmunological parameters at initial radiotherapy such as PIV and SII had significant difference (all *P* < .05, Table 1). Notably, the levels of PIV and SII in patients of DH group were higher than those in NH group (all *P* < .001, Table 1).

- Multivariate logistic regression analysis for risk factors impacting the prognosis in patients with ORNJ

The investigators conducted an unconditional logistic regression analysis utilizing significant factors derived from the univariate analysis. Its results demonstrated that diabetes mellitus, CCI, ASA score, primary tumour site, T stage, ORNJ grade at initial diagnosis, PIV and SII at initial RT were detected as risk factors associated with poor prognosis (delayed healing) in ORNJ patients (all *P* < .05, Table 2).

## Discussion

In this single-institution retrospective investigation, totally 106 patients with clinical or radiographic signs of ORNJ were identified and graded using the adopted modified Glanzmann and Gratz grading system (11). Stabilization of the disease that characterized by chronic persistent non‑progressive lesions (Glanzmann and Gratz type 2) was observed in 55.7% of cases. ORNJ is a well-documented irradiation-induced toxic effect with a varying age and sex distribution in the literature (4,6,13). This may be attributed to the diversities in the study populations, length of follow-up, and in the selection of the location (14). In this study, the cohort’s mean age was 66.2 ± 9.6 years with age range 28-86 years and the male-to-female ratio was 1.6:1 (65 males, 41 females), probably reflecting the different oral care habits.

In our investigation, the predominance of oral cancer (77.8%, 21/27) compared with pharyngeal cancer (22.2%, 6/27) in the occurrence of ORNJ was noted. The reason being oral cancers are approximately four times more likely to develop ORNJ than pharyngeal cancers could due to the greater incidence and prevalence in region that investigated, which was similar to the retrospective study carried out by Kuhnt and Chen 2016 (15,16). The implementation of radiotherapy in the multimodal treatment of head and neck cancer has greatly improved survival rates. In some patients, however, this benefit comes at the potential expense of the tissue surrounding the primary site of malignancy. Osteoradionecrosis is a debilitating complication of radiation therapy and mostly happens in the mandible but seldom occurs in other maxillofacial bones (17). The most common disease site in the study population was mandible (91.5%, 97/106), followed by maxilla (8.5%, 9/106) in our study. The mandible appears to be the most commonly affected osseous structure. This is postulated to be secondary to the relatively poor vascularity within this region as well as local factors including thin mucosal soft tissue coverage, added mechanical stress and remodeling within this region due to forces of mastication, and concomitant dental or periodontal disease (18).

Some other important factors are worth thoroughly pondering, despite they only showed differences that were not statistically significant, e.g., tooth extractions (*P* = .055) and radiation dose (*P* = .422). The association between post-RT extraction and the increased incidence of ORNJ has been fully confirmed, but there remains uncertainty regarding the influence of pre-RT (5,19-21). In previous studies, another significantly increased risk of ORNJ was observed in patients receiving high doses (>60-75 Gy) of irradiation (22,23). Chen *et al*. discovered that a total radiation dose to the primary site of ≥ 75 Gy was an independent factor associated with ORNJ in 1,692 oral cancer patients (16). The MD Anderson Head and Neck Cancer Symptom Working Group declared that most patients with ORNJ have a V44 ≥ 42% and V58 ≥ 25% in patients with oropharyngeal cancer using recursive partitioning analysis in a matched case control study (23).

Bone lysis can occur as early as four hours after irradiation, however, the primary cause of ORNJ seems to be the radiation-induced damage to the endothelial cells. This gives rise to gradual microvascular injury, blockage of blood vessels, which ultimately deteriorates bone turnover due to insufficient oxygen and nutrients (24). Enriched tissue oxygenation is indispensable for all phases of bone repair and regeneration, so hyperbaric oxygen therapy has been suggested as a treatment for radiation tissue injury (late-stage in particular) based on the capacity to improve the blood supply to these tissues (25). Hence, bone growth must be induced by a number of cell types and intracellular, and extracellular molecular signaling pathways. Additionally, pro-inflammatory chemokines, cytokines, recruited multiple immune cells are believed to be relevant with the development of ORNJ through activating a persistent chronic inflammatory phase (26). In recent years, prognostic indexes like PIV, SII, and so on merge varied indicators from the whole blood count and are increasingly recognized as indirect and practical tools for assessing overall inflammation levels in the body (27). Our study explored the correlation associated with ORNJ prognosis using PIV and SII, and the levels of PIV and SII in delayed healing patients were significantly higher than those in normal healing patients (all *P* < .001). In addition, PIV and SII at initial RT were detected as risk factors associated with poor prognosis in patients with ORNJ (all *P* < .05).

Nevertheless, this present study is limited by its retrospective nature, small sample size, and relatively short duration of follow-up.

In conclusion, we preliminarily investigated and comparably analyzed demographic and clinicopathological materials, tumour-and ORNJ-related data between the normal wound healing and delayed wound healing cohorts. More importantly, PIV as well as SII embodying the overall inflammation levels were found to be related to poor prognosis in patients with ORNJ for the first time. Apart from that, diabetes mellitus, CCI, ASA score, primary tumour site, T stage, ORNJ grade at initial diagnosis were also as predictors associated with the poor clinical outcome. Future studies with larger sample sizes, multicentric prospective clinical trials, and longer follow-up data will be crucial to verify our findings.

## Figures and Tables

**Table 1:** Univariate analysis for risk factors of prognostic outcomes in patients with newly diagnosed ORNJ (n = 106).

Variables		No. (%)		Statistics	p value
		NH group (n = 79)	DH group (n = 27)		
Age, yr	Mean (SD)	69 (8)	65 (10)	1.35	0.265
	Median (Q_L_, Q_U_)	65 (28, 86)	66 (30, 86)		
Sex	Male	43 (66.2)	22 (33.8)	0.97	0.325
	Female	36 (87.8)	5 (12.2)		
Smoking history	Former	26 (70.3)	11 (29.7)	2.12	0.125
	Never	53 (76.8)	16 (23.2)		
Alcohol consumption	Former	24 (68.6)	11 (31.4)	1.29	0.289
	Never	55 (77.5)	16 (22.5)		
Oral hygiene condition	Fair	37 (74.0)	13 (26.0)	1.06	0.312
	Poor	42 (75.0)	14 (25.0)		
Diabetes mellitus	Yes	9 (75.0)	3 (25.0)	5.66	0.021*
	No	70 (74.5)	24 (25.5)		
Hypertension	Yes	15 (71.4)	6 (28.6)	2.87	0.096
	No	64 (75.3)	21 (24.7)		
COPD	Yes	1 (16.7)	5 (83.3)	3.99	0.061
	No	78 (78.0)	22 (22.0)		
Hyperthyroidism	Yes	4 (66.7)	2 (33.3)	3.78	0.069
	No	75 (75.0)	25 (25.0)		
Hypothyroidism	Yes	10 (76.9)	3 (23.1)	3.56	0.088
	No	69 (74.2)	24 (25.8)		
CCI	0	33 (75.0)	11 (25.0)	11.02	0.000***
	1	43 (72.9)	16 (27.1)		
	2	3	0		
ASA score	1	17 (73.9)	6 (26.1)	5.75	0.006**
	2	49 (74.2)	17 (25.8)		
	3	13 (76.5)	4 (23.5)		
Tumour site	Oral cavity	64 (75.3)	21 (24.7)	12.34	0.000***
	Oropharynx	10 (76.9)	3 (23.1)		
	Nasopharynx	0	1		
	Hypopharynx	5 (71.4)	2 (28.6)		
T stage	T0—Tx/T1—T2	27 (69.2)	12 (30.8)	6.99	0.003**
	T3—T4	52 (77.6)	15 (22.4)		
N stage	N0—N1/Nx	46 (74.2)	16 (25.8)	4.96	0.052
	N2—N3	33 (75.0)	11 (25.0)		
Dental extraction	Pre-RT	12	4	4.12	0.055
(n = 66, 49/17)	Post-RT	37	13		
Treatment of cancer	Surgery+RT	36	12	0.77	0.781
	Chemotherapy+RT	16	7		
	Surgery+chemotherapy+RT	15	5		
	RT alone	12	3		
Radiation dosage, GyRBE	> 70	37	12	0.95	0.422
	50~70	38	12		
	≤ 50	4	3		
Site of ORNJ	Maxilla	7 (77.8)	2 (22.2)	5.31	0.044*
	Mandible	72 (74.2)	25 (25.8)		
Grade at initial diagnosis	0	15	0	4.90	0.046*
	1	19	6		
	2	40	19		
	3	5	2		
Seroimmunological parameters, listed as Mean ± SD and Median (Q_L_, Q_U_)	PIV at initial RT	463.7 ± 5.1	490.1 ± 9.7	213.40	0.000***
		437.8 (285.8, 678.3)	471.6 (272.5, 693.1)		
	SII at initial RT	733.5 ± 8.0	775.6 ± 10.2		
		732.2 (573.2, 993.5)	780.3 (582.8, 1036.6)		

**Table 2:** Multivariate logistic regression analysis for risk factors of clinical prognosis in patients with ORNJ.

Predictors	β regression coefficient	Standard error	Wald chi-square	OR (95%CI)	p value
Diabetes mellitus	0.84	0.36	3.58	1.68 (0.21 to 6.35)	0.045*
CCI	0.98	0.46	3.78	1.72 (1.16 to 4.85)	0.042*
ASA score	0.58	0.21	7.62	1.80 (1.19 to 2.72)	0.000***
Tumour site	2.18	0.95	5.68	0.97 (0.50 to 2.99)	0.012**
T stage	1.19	0.49	5.95	3.27 (1.26 to 8.61)	0.008**
Site of ORNJ	0.18	0.10	1.69	0.87 (0.14 to 5.51)	0.099
Grade at initial diagnosis	1.21	0.63	7.66	0.72 (0.11 to 1.04)	0.000***
PIV at initial RT	1.03	0.46	6.36	2.86 (1.29 to 6.51)	0.010**
SII at initial RT	0.85	0.52	9.90	2.39 (1.17 to 5.02)	0.000***
